# ICTV Virus Taxonomy Profile: Filoviridae 2024

**DOI:** 10.1099/jgv.0.001955

**Published:** 2024-02-02

**Authors:** Nadine Biedenkopf, Alexander Bukreyev, Kartik Chandran, Nicholas Di Paola, Pierre B. H. Formenty, Anthony Griffiths, Adam J. Hume, Elke Mühlberger, Sergey V. Netesov (Нетёсов Сергей Викторович), Gustavo Palacios, Janusz T. Pawęska, Sophie Smither, Ayato Takada (高田礼人), Victoria Wahl, Jens H. Kuhn

**Affiliations:** 1 Philipps-University Marburg, Marburg, Germany; 2 The University of Texas Medical Branch at Galveston, Galveston, TX, USA; 3 Albert Einstein College of Medicine, New York, USA; 4 United States Army Medical Research Institute of Infectious Diseases, Fort Detrick, Frederick, MD, USA; 5 World Health Organization, Geneva, Switzerland; 6 Chobanian and Avedisian School of Medicine, Boston University, Boston, MA, USA; 7 Novosibirsk State University, Novosibirsk, Russia; 8 Department of Microbiology, Icahn School of Medicine at Mount Sinai, New York, NY, USA; 9 National Institute for Communicable Diseases of the National Health Laboratory Service, Sandringham-Johannesburg, Gauteng, South Africa; 10 Dstl, Porton Down, Wiltshire, UK; 11 International Institute for Zoonosis Control, Hokkaido University, Sapporo, Japan; 12 National Biodefense Analysis and Countermeasures Center, Fort Detrick, Frederick, MD, USA; 13 Integrated Research Facility at Fort Detrick, National Institutes of Health, Fort Detrick, Frederick, Maryland, USA

**Keywords:** Ebola, filovirid, *Filoviridae*, filovirus, ICTV Report, Marburg virus, orthoebolavirus, orthomarburgvirus, taxonomy

## Abstract

*Filoviridae* is a family of negative-sense RNA viruses with genomes of about 13.1–20.9 kb that infect fish, mammals and reptiles. The filovirid genome is a linear, non-segmented RNA with five canonical open reading frames (ORFs) that encode a nucleoprotein (NP), a polymerase cofactor (VP35), a glycoprotein (GP_1,2_), a transcriptional activator (VP30) and a large protein (L) containing an RNA-directed RNA polymerase (RdRP) domain. All filovirid genomes encode additional proteins that vary among genera. Several filovirids (e.g., Ebola virus, Marburg virus) are pathogenic for humans and highly virulent. This is a summary of the International Committee on Taxonomy of Viruses (ICTV) Report on the family *Filoviridae*, which is available at www.ictv.global/report/filoviridae.

## Virion

**Fig. 1.**
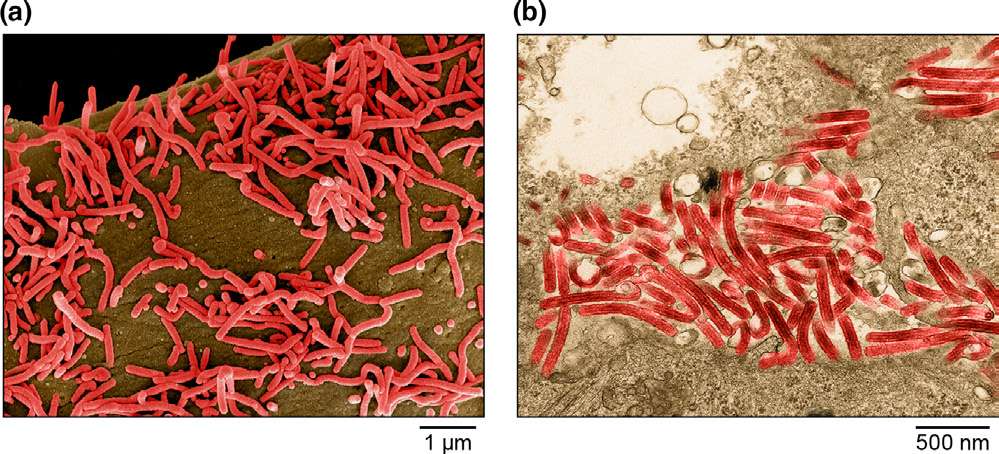
(**a**) Scanning and (**b**) transmission EM images of Marburg virus particles budding from infected Vero E6 cells. Images are colorized for clarity. Courtesy of John G. Bernbaum and Jiro Wada, IRF-Frederick.

Filovirids produce virions that are enveloped and diverse in shape and can be branched, toroid, U- or 6-shaped, or long and filamentous in form (Table 1, Fig. 1). Virions contain ribonucleoprotein (RNP) complexes composed of genomic RNA and, typically, structural proteins, including a nucleoprotein (NP), polymerase co-factor (VP35), transcriptional activator (VP30) and large protein (L). Mammalian filovirid particles also contain an RNP-associated protein (VP24) and a matrix protein (VP40) that form a regular layer beneath the viral envelope. Surface spikes formed by glycoprotein (GP_1,2_) are about 7 nm in diameter and cover the virion surface at roughly 10 nm intervals [1,2].

**Table 1.** Characteristics of members of the family *Filoviridae*

Example	Marburg virus (DQ217792), species *Orthomarburgvirus marburgense*, genus *Orthomarburgvirus*
Virion	Enveloped, variously shaped but predominantly filamentous, typically with a single nucleocapsid
Genome	Approximately 13.1–20.9 kb of linear, negative-sense, non-segmented RNA
Replication	Ribonucleoprotein complexes serve as templates for transcription and replication. Encapsidated antigenomic RNA is a replication intermediate
Translation	From multiple monocistronic 5′-capped and 3′-polyadenylated mRNAs
Host range	Fish (oblaviruses, striaviruses, thamnoviruses), mammals (cuevaviruses, dianloviruses, orthoebolaviruses, orthomarburgviruses), reptiles (tapjoviruses)
Taxonomy	Realm *Riboviria*, phylum *Negarnaviricota*, subphylum *Haploviricotina*, class *Monjiviricetes*, order *Mononegavirales*; >7 genera and >14 species

## Genome

**Fig. 2.**
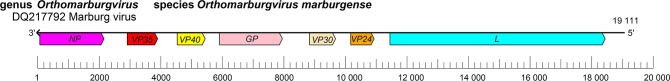
Filovirid genome. *GP*, glycoprotein gene; *L*, large protein gene; *NP*, nucleoprotein gene; *VP30*, transcriptional activator gene; *VP24*, RNP-associated protein gene; *VP35*, polymerase co-factor gene; *VP40*, matrix protein gene.

Filovirid genomes (Fig. 2) are about 13.1–20.9 kb and lack a 5′-cap or 3′-poly(A) tail. Terminal leader and trailer sequences contain the replication and transcription promoters. Six to ten ORFs are flanked by 3′- and 5′-terminal non-coding regions that contain transcription initiation and termination sites. Five ORFs are shared among all filovirids and encode homologous structural proteins (NP, VP35, GP_1,2_, VP30, L). Cuevaviruses and orthoebolaviruses use co-transcriptional editing to express non-structural proteins [1,3].

## Replication

Filovirions attach to cell surface receptors or attachment factors and enter endosomes in which they engage intracellular receptors. pH-dependent fusion with late endosomes releases virion RNP complexes into the cytoplasm. The virus RNP directs both RNA genome replication and gene transcription. Virus proteins are translated from mRNAs that are synthesized by successive, polar transcription from RNP complexes containing genomic RNA. Replication occurs in the cytoplasm through the synthesis of RNP complexes containing antigenomes that are templates for genomic RNA production. Replication and transcription enzymes include L and VP35. VP30 serves as a transcription enhancer and acts at different gene start sites. Whereas orthoebolaviral and cuevaviral VP30 mediates transcription initiation at the *NP* gene start site, orthomarburgviral VP30 mediates transcription reinitiation at the *GP* gene start site. VP30’s function for other filovirids is less defined. In the case of mammalian filovirids, virion assembly, including acquisition of the lipid envelope containing GP_1,2_, occurs by VP40-mediated budding at the plasma membrane [1,4].

## Taxonomy

Current taxonomy: ictv.global/taxonomy. The family *Filoviridae* is included in the negarnaviricot order *Mononegavirales*. Filovirids are most closely related to mononegaviral paramyxovirids, pneumovirids and sunvirids [5].

## Resources

Full ICTV Report on the family *Filoviridae*: www.ictv.global/report/filoviridae.

## Abbreviations

*GP*: glycoprotein gene
GP_1,2_: glycoprotein
L: large protein
NP: nucleoprotein
ORF: open reading frame
RdRP: RNA-directed RNA polymerase
RNP: ribonucleoprotein
VP: viral protein
VP24: RNP-associated protein
VP30: transcriptional activator
VP35: polymerase co-factor
VP40: matrix protein
